# New 5-Aminotetrazole-Based Energetic Polymers: Synthesis, Structure and Properties

**DOI:** 10.3390/ma15196936

**Published:** 2022-10-06

**Authors:** Gennady T. Sukhanov, Konstantin K. Bosov, Yulia V. Filippova, Anna G. Sukhanova, Irina A. Krupnova, Ekaterina V. Pivovarova

**Affiliations:** Laboratory for Chemistry and Technology of High-Energy Azoles, Institute for Problems of Chemical and Energetic Technologies, Siberian Branch of the Russian Academy of Sciences (IPCET SB RAS), Biysk 659322, Russia

**Keywords:** energetic polymers, 5-aminotetrazole, GAP, nucleophilic substitution, nitration

## Abstract

An N-glycidyl-5-aminotetrazole homopolymer was synthesized herein by nucleophilic substitution of 5-aminotetrazole heterocycles for chlorine atoms in poly-(epichlorohydrin)-butanediol. Copolymers of N-glycidyl-5-aminotetrazole and glycidyl azide with a varied ratio of energetic elements were synthesized by simultaneously reacting the 5-aminotetrazole sodium salt and the azide ion with the starting polymeric matrix. The 5-aminotetrazole-based homopolymer was nitrated to furnish a polymer whose macromolecule is enriched additionally with energy-rich terminal ONO_2_ groups and nitrate anions. The structures of the synthesized polymers were characterized by ^1^H and ^13^C NMR and IR spectroscopies, elemental analysis and gel-permeation chromatography. The densities were experimentally measured, and thermal stability data were acquired by differential scanning calorimetry. The insertion of aminotetrazole heterocycles into the polymeric chain and their modification via nitration provides an acceptable thermal stability and a considerable enhancement in density and nitrogen content compared to azide homopolymer GAP. By the 1.3-dipolar cycloaddition reaction, we demonstrated the conceptual possibility of preparing spatially branched, energy-rich polymeric binders bearing 5-aminotetrazole and 1,2,3-triazole heterocycles starting from the plasticized azide copolymers. The presence of the aforesaid advantages makes the reported polymers attractive candidates for use as a scaffold of energetic binders.

## 1. Introduction

Energetic polymers are an essential class of binders when developing and creating advanced, high-performance, energetic condensed systems of composite type [1,2].

From among the energetic polymers, glycidyl azide polymer (GAP) is one of the most common and widely studied [3,4]. The structure of GAP represents a linear polyester macromolecule whose side chain carries explosophoric azidomethyl groups. Because of the acceptable density level [5], good thermal stability [6,7], positive enthalpy of formation [8] and good compatibility with constituents of energetic materials [9,10,11,12], the said azido polymer is one of the most promising alternatives to inert polymeric binders (such as HTPB) to achieve a higher energetic performance of systems based thereon [13,14,15].

That said, despite having positive characteristics, GAP is not free of drawbacks that hinder or restrict its practical use in energy-rich materials [16,17,18]. To overcome the existing drawbacks, different authors are conducting research on modifying the structural arrangement of the azido polymer in order to create novel GAP-based energetic polymers with improved energetic and performance characteristics [19,20,21,22,23,24,25,26,27].

In recent time, tetrazole heterocycles, among the series of explosophoric functional groups, have aroused considerable interest in the chemistry of high-energy materials, as well as in the development of energetic polymers [28,29,30,31,32,33,34,35,36,37,38,39]. Tetrazoles are a unique class of high-nitrogen, aromatic molecular cages that combine a high positive heat of formation and density and good thermal stability, together with a low toxicity and susceptibility to mechanical stimuli [40,41].

High-molecular compounds functionalized by tetrazole derivatives demonstrate excellent energetic characteristics, high thermal stability and resistance to mechanical impacts—which makes them promising candidates for application in energetic formulations of different purposes [28,29,30,31,32,33,34,35,36,37,38,39].

In particular, 5-Aminotetrazole (5-AT) is among the most high-nitrogen, commercially available, starting tetrazole synthons for the synthesis of a broad array of energetic materials [42,43,44,45,46]. The 5-AT molecule is made up of linearly conjugated, heterocyclic N–N, N=N, C–N and C=N bonds and an exocyclic NH_2_ group and contains 82.3% nitrogen. Having quite a high energetic potential, 5-aminotetrazole is not sensitive to mechanical stimuli [42]. Moreover, a rather reactive exocyclic amino group being present in the tetrazole heterocycle offers opportunities for chemical modification of the group and for insertion of additional explosophoric groups (for example, =N–NO_2_, –NH–NO_2_, NO_3_^−^ anion) into the energy-rich molecule of tetrazole, which provides great opportunities for the molecular construction of novel energy-rich compounds based thereon [47,48,49,50,51,52].

Even though a considerable number of various promising energetic polymers structurally containing tetrazole moieties have been devised to date [10], no studies have been performed so far on the synthesis of energetic polymers based on the glycidyl azide polymer, functionalized partially or completely by a commercially available, high-nitrogen 5-aminotetrazole heterocycle, and of their nitrated derivatives. Thus, with the aim of combining a set of useful properties typical of 5-AT and the energetic merits of GAP, the present study synthesized and evaluated key characteristics of new glycidyl polymers that carry 5-aminotetrazole (p-GAT) units and consolidate explosophoric moieties, such as aminotetrazole heterocycles and N_3_ groups (p-(GAT-*co*-GA)), within the single macromolecule. Moreover, a p-GAT-N polymer structurally containing additional oxidizing elements such as ONO_2_ groups and nitrate anions was synthesized herein by nitration of the p-GAT tetrazole homopolymer. Using p-(GAT-*co*-GA) plasticized azide copolymers, we examined rheokinetic and viscosity relationships and demonstrated the feasibility of preparing energy-rich polymeric binders cross-linked with 1,2,3-triazole heterocycles. The structures of all the resultant compounds were characterized by a set of spectroscopic methods (^1^H, ^13^C NMR and IR spectroscopies) and elemental and GPC analyses. The basic characteristics of the synthesized polymers (density, nitrogen content, decomposition temperature) were measured.

## 2. Materials and Methods

The starting chlorine polymer p-ECH-BD and GAP were prepared by the reported procedures [23], and sodium 5-aminotetrazolate, 1,2,3-tris(prop-2-ynyloxy)propane (TPEG) and N-(2-hydroxyethyl)tetrazole (HET) were prepared by the procedures reported in [53,54,55], respectively. All the other chemicals used were purchased from commercial suppliers and employed as received. The IR spectra of the synthesized polymers were recorded on a Simex FT-801 IR-Fourier spectrometer. ^1^H and ^13^C NMR spectra were taken on a Bruker AV-400 (Bruker Corporation, USA) at 400 MHz and 100 MHz, respectively, by using DMSO-d6 signals (δ 2.5 ppm for ^1^H and 39.5 ppm for ^13^C) as internal standard. Elemental analysis of the polymers was performed on a FlashEA1112 CHNS analyzer (Thermo Fisher Scientific Inc., Milan, Italy). The residual chlorine content was quantified on a Multi EA 4000 Cl analyzer (Analytik Jena AG, Jena, Germany). The molecular weight of the polymers was measured by gel-permeation chromatography (GPC) on an LC-20 Prominence high-performance liquid chromatograph (Shimadzu, Japan) with a series of three 300 × 7.5 mm ResiPore columns and a refractive index detector, 3 µm filler particle size (multiparous type) and 7.5 × 50 mm ResiPore Guard pre-columns (Agilent Technologies, Santa Clara, CA, USA). For calibration, polyethylene glycol (PEG) standards with a narrow molecular-weight distribution (M_w_/M_n_ = 1.00−1.09; Agilent Technologies, Santa Clara, CA, USA) were utilized. Eluent was: DMF for GPC (PanReac AppliChem, Darmstadt, Germany) with added LiBr (0.1% concentration). Chromatographic conditions were: 25 °C thermostat temperature, 1.0 mL/min volumetric elution flowrate, 100 μL sample injection volume, 30 g/L and sample concentration. The densities of the synthesized samples were experimentally measured on an AccuPyc II 1340 V1.05 helium pycnometer (Micromeritics, USA). Thermal stability was measured on a DSC 822e (Mettler Toledo, Zurich, Switzerland) instrument at a linear heating rate of 10 °C/min in the range of 20–400 °C under nitrogen. The viscosity measurements were performed on a Brookfield LV DV-II viscometer with a cone–plate system and a Brookfield CP-52 spindle (Brookfield, WI, USA). The temperature during the experiments was controlled by a TC-150AP circulating bath (Brookfield, WI, USA).

CAUTION! All the azido- and tetrazole-containing compounds and the nitrated derivatives thereof are potentially explosion-hazardous energetic materials, though no hazards were observed while synthesizing and handling these compounds. Nonetheless, additional careful measures of precaution are required when dealing with these compounds (grounded equipment, Kevlar^®^ gloves, Kevlar^®^ sleeves, face shield, leather lab coat, ear plugs and protective shield during the reaction).

**Preparation of Glycidyl 5-Aminotetrazole Polymer (p-GAT).** Into a three-neck flask (100 mL) equipped with a magnetic stirrer, a thermometer and a reflux condenser with a calcium chloride drying tube was poured DMF (40 mL), and p-ECH-BD (9.25 g, 100 mmol) was added with stirring. The whole was heated to 130 °C, afterwards sodium 5-aminotetrazolate (16.07 g, 150 mmol) was added. The resultant reaction mixture was stirred at 130 °C until the Beilstein test showed no chlorine. Upon reaction completion, the mixture was cooled to room temperature, and the precipitate as salts was collected by filtration. The polymeric solution was poured into isopropanol with vigorous stirring, and the precipitated product was separated by filtration. The resultant polymer was additionally purified by re-precipitation from a water–acetone mixture (water/acetone = 30/70 wt.%) into isopropanol. The polymer concentration in the water–acetone mixture was 25 wt.%. Following several washings with isopropanol and drying until constant weight at 80 °C in vacuo, the p-GAT homopolymer (7.32 g, 51.9%) was obtained as a white powder. IR spectrum (KBr), ν, cm^−1^ was: 3334–3238 (NH_2_), 2937–2880 (CH, CH_2_ of the polymeric chain), 1631–1476 (C=N), 1551–1360, 1308–757 (NCN, NN, CN of the tetrazole ring), 1114 (C–O–C of the polymeric chain). ^1^H NMR (400 MHz, DMSO-d_6_), δ, ppm was: 6.62 (s, 2H, NH_2_ N1-isomer of 5-AT), 5.99 (s, 2H, NH_2_ N2-isomer of 5-AT), 4.51–3.30 (proton signal region: –CH–O, –CH_2_–O groups of the chain and –CH_2_ related to the 5-AT heterocycle), 1.52 (s, 2H, –CH_2_ of BDO). ^13^C NMR (100 MHz, DMSO-d_6_), δ, ppm was: 167.45 (C–NH_2_ N2-isomer of 5-AT), 156.52 (C–NH_2_ N1-isomer of 5-AT), 78.15–77.17 (–O–CH), 71.86–68.12 (–O–CH_2_), 53.29 (–CH_2_–N of 5-AT), 26.25–25.90 (–CH_2_ of BDO). Elemental analysis was: calcd (%) for C_4.2_H_7.5_N_5.1_ (146.49): C 34.76; H 5.15; N 48.29; found (%): C 35.01; H 5.18; N 47.95.

**Nitration of Glycidyl 5-Aminotetrazole Polymer (p-GAT-N).** The p-GAT-N polymer was prepared by dissolving the p-GAT homopolymer (3.67 g, 26 mmol) in excess concentrated HNO_3_ (65% conc., 26 mL) with stirring at room temperature. The solution was heated to 96–100 °C and stirred for 10 min. The cooled reaction mass was poured into a great amount of ice and washed with distilled water, until neutral wash waters was achieved, and with isopropanol. The filtered product was dried at 50 °C in vacuo until constant weight to furnish a light-yellow powder (1.58 g, 29.7%). IR spectrum (KBr), ν, cm^−1^ was: 3354–3149 (NH_2_), 2931–2878 (CH, CH_2_ of the polymeric chain), 1716, 1581, 1253 (ONO_2_), 1623 (C=N), 1505, 1385, 1314 (NO_3_^−^), 1444–710 (NCN, NN, CN of the tetrazole ring), 1120 (C–O–C of the polymeric chain) ^1^H NMR (400 MHz, DMSO-d_6_), δ, ppm was: 8.90 (s, 6H, NH+NH_2_ N1-isomer, NH+NH_2_ N2-isomer), 4.80–3.40 (proton signal region: –CH–O, –CH_2_–O groups of the chain and –CH_2_ related to the 5-AT heterocycle), 1.51 (s, 2H, –CH_2_ of BDO). ^13^C NMR (100 MHz, DMSO-d_6_), δ, ppm was: 153.62 (C–NH_2_ N2-isomer of 5-AT), 152.50 (C–NH_2_ N1-isomer of 5-AT), 79.31–76.15 (–O–CH), 71.60–66.63 (–O–CH_2_), 53.56 (–CH_2_–N of 5-AT), 25.89 (–CH_2_ of BDO). Elemental analysis was: calcd (%) for C_4.2_H_8.4_N_6.0_ (208.83): C 24.16; H 4.05; N 40.38; found (%): C 25.33; H 4.88; N 40.10.

**Preparation of Glycidyl 5-Aminotetrazole and Glycidyl Azide Copolymers (p-(GAT-co-GA)).** Into a three-neck flask (100 mL) fitted with a magnetic stirrer, a thermometer and a reflux condenser with a calcium chloride drying tube was poured DMF (40 mL), and p-ECH-BD (9.25 g, 100 mmol) was added with stirring. The whole was heated to 130 °C; afterwards, sodium 5-aminotetrazolate (14.46 g, 135 mmol), NH_4_Cl (0.80 g, 15 mmol) and NaN_3_ (0.98 g, 15 mmol) were added successively. The stirring was continued at 130 °C until the Beilstein test showed no chlorine. Upon reaction completion, the reaction mass was cooled down, and the precipitate was collected by filtration. The polymeric solution was poured into isopropanol with vigorous stirring, and the precipitated product was separated by filtration. The resultant polymer was additionally purified by re-precipitation from a water–acetone mixture (water/acetone = 30/70 wt.%) into isopropanol. The polymer concentration in the water–acetone mixture was 25 wt.%. Several washings with isopropanol and drying at 50–55 °C in vacuo until constant weight afforded the p-(GAT-*co*-GA)-14 copolymer as a cream-colored powder (7.57 g, 56.9%). IR spectrum (KBr), ν, cm^−1^ was: 3336–3242 (NH_2_), 2930–2882 (CH, CH_2_ of the polymeric chain), 2104 (N_3_), 1632–1475 (C=N), 1550–1364, 1307–759 (NCN, NN, CN of the tetrazole ring), 1119 (C–O–C of the polymeric chain). ^1^H NMR (400 MHz, DMSO-d_6_), δ, ppm was: 6.62 (s, 2H, NH_2_ N1-isomer of 5-AT), 5.99 (s, 2H, NH_2_ N2-isomer of 5-AT), 4.52–3.30 (proton signal region: –CH–O, –CH_2_–O groups of the chain and –CH_2_ associated with N_3_ and the 5-AT heterocycle), 1.52 (s, 2H, –CH_2_ of BDO). ^13^C NMR (100 MHz, DMSO-d_6_), δ, ppm was: 167.46 (C–NH_2_ N2-isomer of 5-AT), 156.52 (C–NH_2_ N1-isomer of 5-AT), 78.19–77.19 (–O–CH), 71.00–69.14 (–O–CH_2_), 53.30 (–CH_2_–N of 5-AT), 51.37 (–CH_2_–N_3_) 26.26–25.90 (–CH_2_ of BDO). Elemental analysis was: calcd (%) for C_4.1_H_7.1_N_4.7_ (139.19): C 35.03; H 5.17; N 47.50; found (%): C 35.42; H 5.23; N 46.98.

The p-(GAT-*co*-GA)-22 copolymer was synthesized and purified under the same conditions as for p-(GAT-*co*-GA)-14, except the fact that to the solution of p-ECH-BD (9.25 g, 100 mmol) in DMF (40 mL) were added sodium 5-aminotetrazolate (13.39 g, 125 mmol), NH_4_Cl (1.34 g, 25 mmol) and NaN_3_ (1.63 g, 25 mmol). The result was the p-(GAT-*co*-GA)-22 copolymer as a cream-colored powder (8.00 g, 61.7%). IR spectrum (KBr), ν, cm^−1^ was: 3329–3242 (NH_2_), 2923–2877 (CH, CH_2_ of the polymeric chain), 2103 (N_3_), 1636–1474 (C=N), 1550–1363, 1305–758 (NCN, NN, CN of the tetrazole ring), 1119 (C–O–C of the polymeric chain). ^1^H NMR (400 MHz, DMSO-d_6_), δ, ppm was: 6.64 (s, 2H, NH_2_ N1-isomer of 5-AT), 6.00 (s, 2H, NH_2_ N2-isomer of 5-AT), 4.51–3.12 (proton signal region: –CH–O, –CH_2_–O groups of the chain and –CH_2_ associated with N_3_ and the 5-AT heterocycle), 1.52 (s, 2H, –CH_2_ of BDO). ^13^C NMR (100 MHz, DMSO-d_6_), δ, ppm was: 167.48 (C–NH_2_ N2-isomer of 5-AT), 156.52 (C–NH_2_ N1-isomer of 5-AT), 78.28–77.20 (–O–CH), 71.86–69.12 (–O–CH_2_), 53.30 (–CH_2_–N of 5-AT), 51.38 (–CH_2_–N_3_) 26.26–25.90 (–CH_2_ of BDO). Elemental analysis was: calcd (%) for C_4.0_H_7.0_N_4.6_ (136.40): C 35.22; H 5.18; N 46.93; found (%): C 35.76; H 5.26; N 46.23.

The p-(GAT-*co*-GA)-56 copolymer was synthesized under the same temperature–time conditions as for p-(GAT-*co*-GA)-14, except for the fact that to the solution of p-ECH-BD (9.25 g, 100 mmol) in DMF (40 mL) were added sodium 5-aminotetrazolate (9.63 g, 90 mmol), NH_4_Cl (3.21 g, 60 mmol) and NaN_3_ (3.90 g, 60 mmol). Upon reaction completion, the reaction mass was cooled down, the precipitate was collected by filtration and DMF was evaporated at 85–90 °C in vacuo. The residue was mixed with acetone in a ratio of 50/50 wt.%, and the resulting suspension was passed through a Captiva premium syringe filter having a polytetrafluoroethylene membrane and a pore size of 0.45 µm (Agilent Technologies, USA). The polymeric solution was poured into water with vigorous stirring. Several washings with water and drying at 90 °C in vacuo until constant weight furnished the p-(GAT-*co*-GA)-56 copolymer as a high-viscosity mass (8.25 g, 71.0%). IR spectrum (film), ν, cm^−1^ was: 3331–3189 (NH_2_), 2922–2876 (CH, CH_2_ of the polymeric chain), 2095 (N_3_), 1629–1477 (C=N), 1547–1350, 1275–757 (NCN, NN, CN of the tetrazole ring), 1105 (C–O–C of the polymeric chain). ^1^H NMR (400 MHz, DMSO-d_6_), δ, ppm was: 6.61 (s, 2H, NH_2_ N1-isomer of 5-AT), 6.00 (s, 2H, NH_2_ N2-isomer of 5-AT), 4.52–3.14 (proton signal region: –CH–O, –CH_2_–O groups of the chain and –CH_2_ associated with N_3_ and the 5-AT heterocycle), 1.53 (s, 2H, –CH_2_ of BDO). ^13^C NMR (100 MHz, DMSO-d_6_), δ, ppm was: 167.50 (C–NH_2_ N2-isomer 5-AT), 156.51 (C–NH_2_ N1-isomer of 5-AT), 78.49–77.32 (–O–CH), 71.89–69.13 (–O–CH_2_), 53.30 (–CH_2_–N of 5-AT), 51.48 (–CH_2_–N_3_) 26.29–25.90 (–CH_2_ of BDO). Elemental analysis was: calcd (%) for C_3.7_H_6.4_N_3.9_ (122.52): C 35.98; H 5.22; N 44.70; found (%): C 36.71; H 5.33; N 43.81.

The p-(GAT-*co*-GA)-75 copolymer was synthesized under the same temperature–time conditions as for p-(GAT-*co*-GA)-14, except for the fact that to the solution of p-ECH-BD (9.25 g, 100 mmol) in DMF (40 mL) were added sodium 5-aminotetrazolate (8.03 g, 75 mmol), NH_4_Cl (4.01 g, 75 mmol) and NaN_3_ (4.88 g, 75 mmol). Upon reaction completion, the mass was cooled down, the precipitate was collected by filtration and the solvent was evaporated at 85–90 °C in vacuo. The residue was mixed with dichloromethane (50 mL), and the resulting suspension was passed through a Captiva premium syringe filter having a polytetrafluoroethylene membrane and a pore size of 0.45 µm (Agilent Technologies, Santa Clara, CA, USA). The polymeric solution was washed thrice with water. After it was dried over MgSO_4,_ and the solvent was evaporated at 80–90 °C in vacuo until constant weight, the p-(GAT-*co*-GA)-75 copolymer was obtained as a viscous mass (6.65 g, 61.2%). IR spectrum (film), ν, cm^−1^ was: 3333–3189 (NH_2_), 2919–2872 (CH, CH_2_ of the polymeric chain), 2091 (N_3_), 1632–1473 (C=N), 1548–1346, 1275–748 (NCN, NN, CN of the tetrazole ring), 1104 (C–O–C of the polymeric chain). ^1^H NMR (400 MHz, DMSO-d_6_), δ, ppm was: 6.62 (s, 2H, NH_2_ N1-isomer of 5-AT), 6.01 (s, 2H, NH_2_ N2-isomer of 5-AT), 4.53–3.14 (proton signal region: –CH–O, –CH_2_–O groups of the chain and –CH_2_ associated with N_3_ and the 5-AT heterocycle), 1.54 (s, 2H, –CH_2_ of BDO). ^13^C NMR (100 MHz, DMSO-d_6_), δ, ppm was: 167.51 (C–NH_2_ N2-isomer of 5-AT), 156.50 (C–NH_2_ N1-isomer of 5-AT), 78.50–77.34 (–O–CH), 71.90–69.14 (–O–CH_2_), 53.35 (–CH_2_–N of 5-AT), 51.50 (–CH_2_–N_3_) 26.72–26.22 (–CH_2_ of BDO). Elemental analysis was: calcd (%) for C_3.3_H_5.6_N_3.3_ (107.60): C 36.49; H 5.26; N 43.17; found (%): C 37.27; H 5.37; N 42.26.

## 3. Results and Discussion

### 3.1. Synthesis and Chemical Structure Analysis

For the synthesis of energetic polymers functionalized by tetrazole heterocycles and/or azido groups, we used poly-(epichlorohydrin)-diol (p-ECH-BD) as the starting polymeric matrix representing an epichlorohydrin polymer with central butanediol units and terminal OH groups and structurally containing reactive functional chlorine atoms capable of being substituted. The p-ECH-BD polymer was prepared by the cation polymerization technique through the epoxy ring-opening of epichlorohydrin (ECH) by an initiating system based on boron trifluoride etherate and 1,4-butanediol (BDO). The polymerization process of ECH was effected under conditions of the activated monomer mechanism [23] that precludes side reactions of the formation of low-molecular cyclic products and provides the best control of molecular weight and polydispersity.

The common method for functionalization of polymer precursors by explosophoric groups is the reaction of nucleophilic substitution of reactive groups (chlorine, bromine, mesylate group) of the polymeric matrix [56,57,58,59]. Here, p-ECH-BD was chemically modified through the nucleophilic substitution by heating the mixed reactants at 130 °C in aprotic high-boiling solvent DMF, which dissolves quite well the starting polymer precursor—the sodium salt of 5-aminotetrazole and sodium azide. The concentration of the chlorine polymer in the chosen solvent, which makes sure that the reaction mixture is homogeneous and the polymer is not deposited by the reaction, was 20 wt.%. To enhance the nucleophilicity, 5-AT was employed in the salt form with Na^+^ cations (sodium 5-aminotetrazolate, Na5-AT). The substitution of chlorine atoms in p-ECH-BD by tetrazole heterocycles and/or the azido group was performed with a 50% excess of the corresponding reactant or their mixture. The degree of substitution reaction was maintained until the high-sensitive Beilstein test [60] showed no organic chlorine in the reaction mixture and until the characteristic signals of the chloromethyl moieties of the starting polymer were absent according to the ^13^C NMR and IR spectroscopic data.

**Scheme 1:**
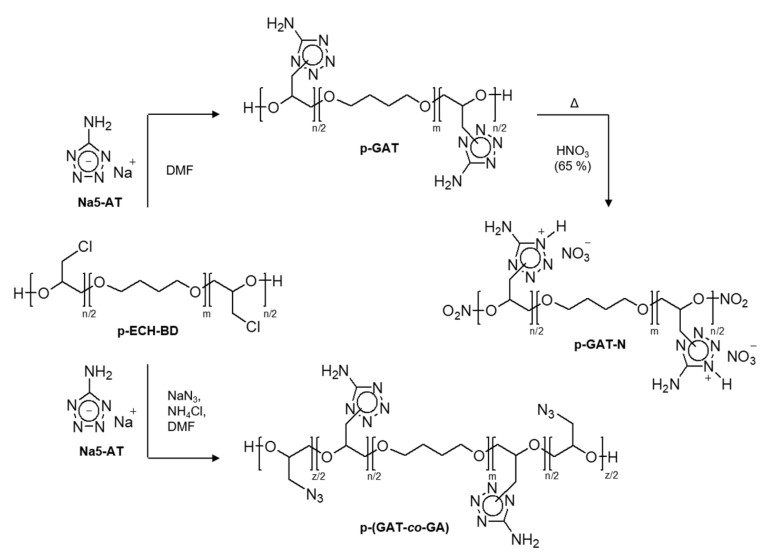
A general synthetic protocol for the p-GAT, p-GAT-N and p-GT-AN polymers and p-(GAT-*co*-GA) copolymers.

Under the aforesaid conditions, the reaction of sodium 5-aminotetrazole and/or the azide ion with the starting polymer precursor took place towards the substitution of chlorine atoms, with an insertion of the respective energy-rich substituents into the macromolecule structure (Scheme 1).

Upon completion of the reaction between sodium 5-AT and p-ECH-BD (12 h at 130 °C), a p-GAT homopolymer containing 5-aminotetrazole heterocycles was obtained, with the degree of substitution of the halogen by aminotetrazole being 99.3%. The degree of substitution was measured from the residual chlorine content of the product by using an analyzer for total chlorine. The chlorine content of the target homopolymer was 0.17%. The polymer yield was about 52% of the theoretical possible. Proceeding from quite a high substitution of the halogen by the tetrazole heterocycles, the temperature–time nucleophilic substitution parameters employed in the synthesis of p-GAT were used for the preparation of the p-(GAT-*co*-GA) azide copolymers.

The 5-Aminoterazole compound and derivatives thereof are known to be capable of reacting with nitric acid to produce energetic salts in which the aminotetrazole heterocycle acts as a cation, while NO_3_^−^ serves as an anion [61,62,63,64,65]. Such high-energy ionic salts offer advantages over non-ionic molecules because of a lower vapor pressure and a higher density [61,62,63,64,65]. Given this, for the structure to be additionally enriched with functional nitrogen–oxygen-bearing groups, the p-GAT homopolymer was subjected to nitration with excess concentrated HNO_3_ (65%). When reacted with the nitrating agent, a polymeric salt structure began to be formed in which the cation was the macromolecule bearing aminotetrazole moieties, while the anion was the nitrate anion (p-GAT-N). Moreover, the terminal OH groups of the precursor transformed into energy-rich ONO_2_ groups. The high-temperature synthesis promotes the progress of a side process associated with thermolysis of the aminotetrazole ring, leading to the ring-opening and formation of the azido form that occurs when 5-AT and its derivatives are exposed to high temperatures [66,67]. The partial thermolysis progress was evidenced by the presence of an exoeffect during the nitration, the appearance of IR signals in the region typical of azido groups and the small yield of the p-GAT-N product.

The p-(GAT-*co*-GA) copolymers of N-glycidyl-5-aminotetrazole and glycidyl azide were synthesized by simultaneously reacting the starting p-ECH-BD polymeric matrix with both 5-AT heterocycles as the sodium salt and sodium azide. The reactivity of the azide ion increased significantly when the azidation reaction was carried out in the presence of compounds that, when reacted with NaN_3_, generate hydrazoic acid salts that are much more soluble in DMF [68]. Concurrently with an improvement in solubility, such salts increase the reactivity of the azide ion [68]. In the synthesis of p-(GAT-*co*-GA) copolymers, we used mixed NaN_3_/NH_4_Cl as the azidizing system, which were taken in an equimolar ratio. A variation in the ratio of Na5−AT and the azidizing system allowed the copolymers to be prepared with a varied ratio of energetic moieties in the macromolecule structure, with a high halogen degree of conversion and a yield above 56% (Table 1).

The high organophilic behavior of the synthesized polymers promoted a decline in their yield when they were treated with organic solvents during their isolation from the reaction system.

To evaluate the structural characteristics, thermal stability and density, we prepared azide homopolymer GAP as a reference sample via the nucleophilic substitution of the p-ECH-BD halogen by the NaN_3_–NH_4_Cl azidizing system under conditions reported in [23].

The structural characteristics of the resultant 5-AT-based polymers were characterized by a set of spectral techniques (NMR and IR spectroscopies) and gel-permeation chromatography.

**Figure 1:**
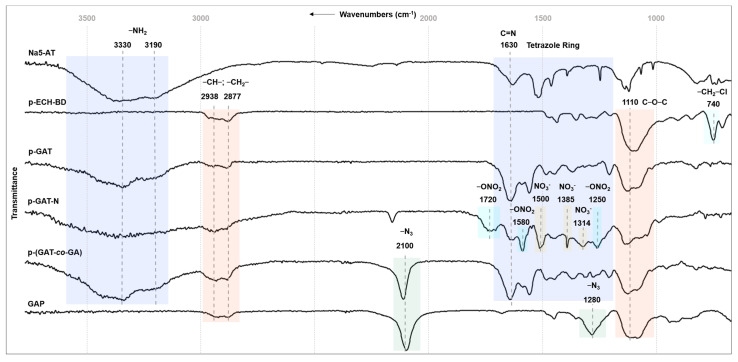
IR spectra of the starting Na5-AT and p-ECH-BD and of target polymers p-GAT, p-GAT-N, p-(GAT-*co*-GA) and GAP.

Figure 1 depicts IR spectra of the starting Na5-AT and p-ECH-BD and of the synthesized polymers. Irrespective of the type of the energy-rich moiety (aminotetrazole heterocycles and/or N_3_ group, nitrate anion) that was incorporated into the starting p-ECH-BD macromolecule or underwent a chemical modification by nitration, the shape and frequency of symmetric vibrations of the ether link (C–O–C) of the polymeric backbone retained within the range of 1104–1119 cm^−1^. The characteristic bands of stretching vibrations of the CH- and CH_2_ groups of the glycidyl chain retained as well for all the synthesized polymers in the range of 2872–2937 cm^−1^. The presence of the said characteristic vibrational bands of the major bonds and groups of the polymeric chain evidences absent destructive processes of the macromolecule during the reactions of nucleophilic substitution and nitration. The absorption bands of stretching vibrations of the CH_2_-Cl bonds of the starting p-ECH-BD at 748 cm^−1^ show up only for the intermediates at shorter substitution reaction times. The IR spectra of the target polymers have no absorption band of chloromethyl groups, suggesting quite a high degree of substitution of the halogen by the corresponding energy-rich moiety.

For compounds p-GAT, p-GAT-N and p-(GAT-*co*-GA), the aminotetrazole heterocycles found in the structure corroborate the IR spectrum to have the most frequency-characteristic intense absorption bands of skeletal vibrations of the ring and the ring-related exocyclic NH_2_ group.

The stretching and stretching–bending skeletal vibrations of the tetrazole heterocycles (NCN, NN, CN) appear near 1000–1700 cm^−1^, intermix with each other and exhibit several intense absorption bands in the ranges of 1068–1084 cm^−1^, 1200–1278 cm^−1^, 1305–1364 cm^−1^, 1438–1477 cm^−1^ and 1550–1637 cm^−1^ [29,44,47,53]. The most intense band among the listed ones is the absorption band near 1629–1637 cm^−1^, which is related to the C=N vibrations of the tetrazole ring [44,53]. The symmetric and asymmetric stretching vibrations of the amino group appear as intense and broadened absorption bands because of hydrogen bonding in the high-frequency region of 3329–3336 cm^−1^ and 3189–3242 cm^−1^, respectively [53]. The present signals of the NH_2_ group indicate that the nucleophilic substitution proceeded to incorporate aminotetrazole heterocycles into the polymeric chain over the endocyclic nitrogen atoms of the ring, not affecting the exocyclic amino group.

For the nitrated derivative (p-GAT-N), in addition to the skeletal vibrations of the tetrazole heterocycles, the IR spectra show characteristic intense absorption bands of the NO_3_^−^ anion at 1505 cm^−1^, 1385 cm^−1^ and 1314 cm^−1^ [62,64,65], and characteristic vibrations of the ONO_2_ groups resulted from the nitration of the terminal OH groups of the starting p-GAT homopolymer: bands at 1717 cm^−1^, 1581 cm^−1^ and 1253 cm^−1^ [29,69,70].

The azido moieties in the structures of azide homopolymer GAP and p-(GTO-*co*-GA) copolymers are identifiable from the present characteristic stretching vibrations of the N_3_ groups appearing at 2097–2104 cm^−1^ [27].

**Figure 2:**
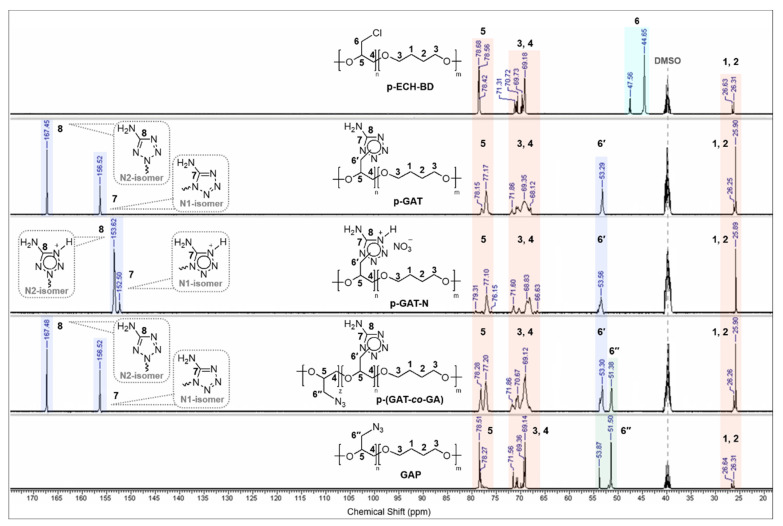
^13^C NMR spectra of p-ECH-BD, p-GAT, p-GAT-N, p-(GAT-*co*-GA) and GAP polymers.

The ^13^C NMR spectra of the resultant polymers (Figure 2) show a resonance of carbon atoms of the glycidyl backbone and butanediol units: carbon atoms of the ester bond at 66.6–71.9 ppm (–CH_2_–O; **3**, **4**) and 76.2–79.3 ppm (–CH–O; **5**), and central carbon atoms of the diol moiety at 25.9–26.6 ppm (–CH_2_–; **1**, **2**). The carbon signals of the chloromethyl groups (**6**) of the starting polymeric matrix (p-ECH-BD) near 44.7–47.6 ppm disappear completely following the nucleophilic substitution by the corresponding energy-rich moiety. The resonance signals of the carbon atoms located in the side chain and related to 5-aminotetrazole heterocycles (**6′**) appear in the spectra at 53.3–53.7 ppm (for p-GAT, p-GAT-N and p-(GAT-*co*-GA)).

The alkylation of 5-aminotetrazole in basic media is known to take place to furnish isomeric derivatives substituted on the heterocyclic N-1 and N-2 atoms [44,71,72,73]. The two characteristic carbon signals present in the ^13^C NMR spectra of the resultant p-GAT and p-(GAT-*co*-GA) polymers indicate two 5-AT isomers present in the macromolecule at 156.5 ppm (C–NH_2_; N-1 isomer **7**) and 167.5 ppm (C–NH_2_; N-2 isomer **8**) [74].

In the spectra of the nitrated p-GAT-based derivative, the carbon signals of the isomers are shifted upfield at 152.5 ppm (C–NH_2_; N-1 isomer **7**) and 153.6 ppm (C–NH_2_; N-2 isomer **8**) because of the ionic nature.

For the azide polymers (GAP and p-(GAT-*co*-GA)), the resonance carbon atom signals of the –CH_2_–N_3_ groups (**6**″) show up at 51.4–51.5 ppm in the ^13^C NMR spectra. The ratio of glycidyl triazolone and glycidyl azide units in the p-(GAT-*co*-GA) copolymers was estimated from the ^13^C NMR spectra of the corresponding samples. The results are summarized in Table 1.

**Figure 3:**
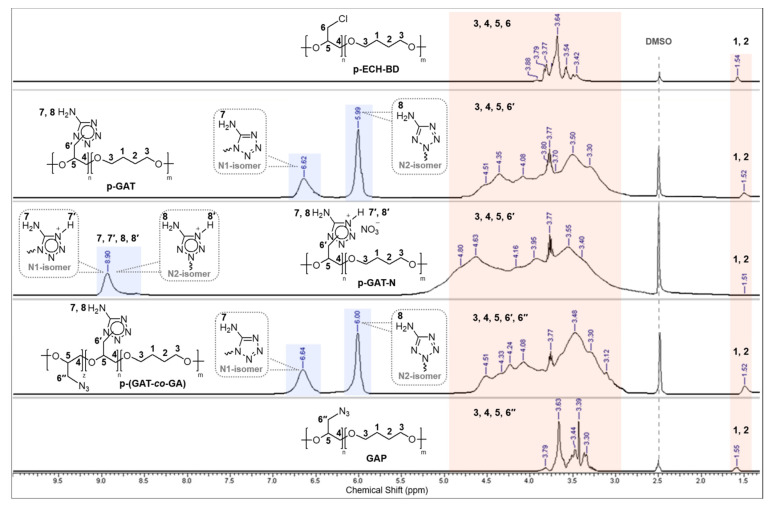
^1^H NMR spectra of p-ECH-BD, p-GAT, p-GAT-N, p-(GAT-*co*-GA) and GAP polymers.

Due to the polymeric nature of the target compounds, the proton signals of the polymeric chain units have a characteristic broadened appearance in the ^1^H NMR spectra (Figure 3). The spectra of all the polymers retain proton signals of the glycidyl diol backbone and overlap over each other and are at between 3.30 ppm and 3.95 ppm (–O–CH_2_; **3**, **4** and –O–CH; **5**). The resonance proton signals of the central units of butanediol were documented separately at 1.52–1.55 ppm (–CH_2_; **1**, **2**). The proton signals of the –CH_2_– groups (**6′**) associated with the endocyclic N-1 and N-2 atoms of the aminotetrazole heterocycle, as well as with the N_3_ groups, show up in the range of 4.08–4.80 ppm (**6**″). Broadened by the hydrogen bonding, the proton singlets of the exocyclic NH_2_ group of the isomeric tetrazole moieties were resonating separately in the downfield for the p-GAT and p-(GAT-*co*-GA) polymers.

In that case, the proton signals of the amino group typical of the N-1 substituted isomeric tetrazole moieties are shifted downfield at 0.61–0.64 ppm relative to the respective proton signals of the NH_2_ group of the N-2 isomeric moieties: 5.99–6.01 ppm (NH_2_; N-2 isomer **8**) and 6.61–6.64 ppm (NH_2_; N-1 isomer **7**) [71,72,73].

The analysis data of the ^1^H NMR spectra of the nitrated p-GAT-N polymer are on par with the known results of the spectral analysis of the low-molecular counterparts, such as 1-methyl- and 2-methyl-5-aminotetrazol-4-ium nitrates [62,65], that have characteristic proton signals of the amino groups near 8.5–9.1 ppm. Because of the polymeric nature and the presence of hydrogen bonds, the proton signals of the NH_2_ groups (**7**, **8**) and the protons of (**7′**, **8′**) isomeric units of the p-GAT-N polymer were recorded as a single broadened signal at 8.9 ppm.

The ratio of the N-1 and N-2 isomeric moieties contained in the polymeric chain structure was determined from the ratio of the integral intensities of the proton signals of the corresponding amino groups in the ^1^H NMR spectra (Table 1). The isomeric composition of the corresponding polymer depends on nucleophilic substitution reaction conditions. Under conditions of no azidizing system (NaN_3_–NH_4_Cl) in the reaction mixture, the halogen was substituted in the polymeric matrix simultaneously on N1 and N-2 of the aminotetrazole heterocycle, whose proportion attained 57% (p-GAT), with the chain containing chiefly N-2 isomeric moieties. The insertion of the azidizing system into the reaction mixture and its successive increase during the synthesis of the corresponding p-(GAT-*co*-GA) copolymer were likely to promote an increase in the reaction medium basicity, thereby increasing the accessibility of the most basic reactive site in the 5-AT molecule, i.e., the nitrogen atom at the N-1 position of the heterocycle. That said, the portion of N-1 substituted moieties increased from 44% (p-(GAT-*co*-GA)-14) to 51% (p-(GAT-*co*-GA)-75) relative to the N-2 isomers.

The molecular-weight characteristics of the synthesized polymers were evaluated by GPC using an HPLC chromatograph equipped with a refractive index detector. Polyethylene glycol samples with the known molecular weights and a low polydispersity were utilized as the calibration standards. It is seen from the findings listed in Table 1 that the molecular weight (M_w_) of the polymers varied in a range of 1300–5500 g/mol and depended considerably on the 5-AT unit content in the structure. The p-(GAT) aminotetrazole-bearing homopolymer had the highest M_w_. A decrease in the portion of aminotetrazole moieties from 86 to 25 mol% in the macromolecule structure resulted in a decline in the molecular weights of the p-(GAT-*co*-GA) copolymers from 4000 g/mol to 1600 g/mol. The GAP azide polymer containing no 5-AT units has a M_w_ of about 1300 g/mol. That said, all the polymers exhibit quite a moderate level of polydispersity (M_w_/M_n_): 1.19–1.45. The tendency towards an increase in the content of aminotetrazole moieties in the polymer structure (transition from GAP to p-(GAT-*co*-GA) and p-(GAT)) can be caused by increased intra- and intermolecular hydrogen bonding, typical of tetrazole polymers, and by enhanced self-association of molecular chains, while the amino group present in the tetrazole heterocycle only amplified these effects [75,76]. This is reflected in the GPC chromatograms: the chromatograms have a broadened appearance for the samples with a high content of aminotetrazole moieties.

It has been established from the results of spectral studies and GPC analysis that the reaction between the starting chlorine polymeric matrix p-ECH-BD and Na-5-AT and/or NaN_3_ under the used nucleophilic substitution conditions provides an insertion of aminotetrazole heterocycles and/or N_3_ groups into the polymeric chain in place of the halogen. The substitution of aminotetrazole for chlorine atoms proceeded at the N-1 and N-2 positions of the heterocycle, and the ratio of the isomeric moieties was dependent on the reaction conditions. The ratio of the structural elements in the single molecule and the level of molecular-weight characteristics of the polymers are controlled by varying the ratio of the starting reactants. A p-GAT-N polymer structurally bearing additional energy-rich moieties (ONO_2_ groups, NO_3_^−^ anion) was prepared by nitration of the p-GAT sample. No variations in the polymeric backbone were observed during the chemical modification (nucleophilic substitution, nitration).

### 3.2. Thermal Stability, Nitrogen Content and Density

The high thermal stability, high nitrogen content and the high density level are among the key properties of efficient energetic materials [29]. The thermal stability of the synthesized polymers was measured by differential scanning calorimetry (DSC) in a temperature range of 20–400 °C.

**Figure 4:**
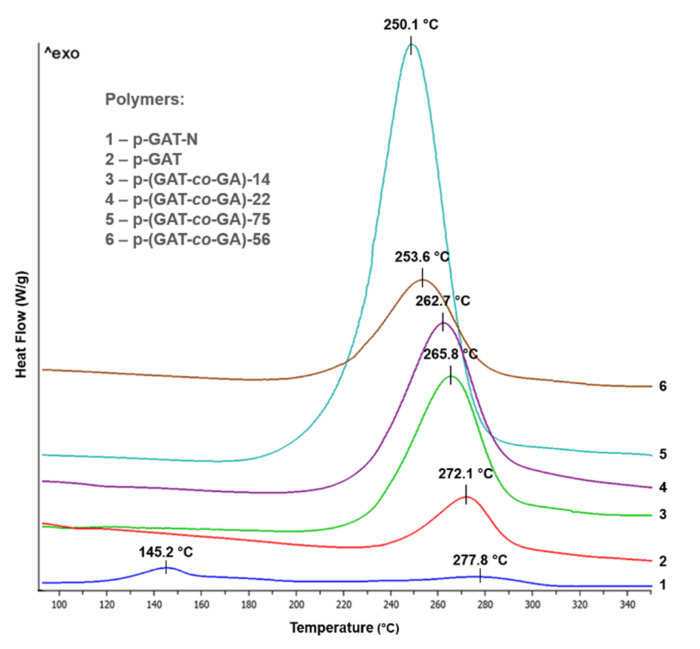
A DSC image of the p-GAT and p-GAT-N polymers and of p-(GTO-*co*-GA) copolymers.

The 5-aminotetrazole-based homopolymer has a high thermal stability, and its thermal decomposition induced by exothermal decomposition of tetrazole moieties takes place in one stage. The DSC image of p-GAT shows no changes up to 220 °C. Above that temperature, a considerable exothermal effect occurs that corresponds to the onset of thermal decomposition at 243 °C. The temperature peak of the single-stage decomposition of the p-GAT homopolymer is documented at 272 °C (Table 2, Figure 4).

The modification of the p-GAT polymer structure through its nitration alters the decomposition nature (Figure 4). For the p-GAT-N polymer, the decomposition takes place in two stages. The first stage shows a broadened exothermal effect with an onset temperature of 122 °C and a temperature peak of 145 °C. The thermal decay at this stage seems to be induced by decomposition of explosophoric moieties, such as nitrate anion and terminal nitrate groups of the polymeric chain. The second high-temperature stage shows a thermal decomposition of the aminotetrazole moieties of the macromolecule, with an onset effect at 241 °C. The temperature peak at this stage is at 278 °C.

The variation in the ratio of the energetic moieties (GAT and GA) within the single polymeric matrix afforded copolymers in different aggregate states, ranging from the viscous-flow ones at room temperature—(p-(GAT-*co*-GA)-56 and p-(GAT-*co*-GA)-75)—to the samples as amorphous loose powders—(p-(GAT-*co*-GA)-14 and p-(GAT-*co*-GA)-22).

The thermal decomposition of the p-(GAT-*co*-GA) copolymers appears to have a mechanism similar to the p-GAT homopolymer, proceeds in one stage and is evoked by a simultaneous exothermal decomposition of both the aminotetrazole moieties and the N_3_ groups. Along with that, the insertion of aminotetrazole moieties into the polymeric chain structure makes the compound more resistant to a temperature exposure. The onset temperature of decomposition of the copolymers when p-(GAT-*co*-GA)-75 transits to p-(GAT-*co*-GA)-14 is shifted towards higher values, from 220 to 233 °C, respectively (Table 2, Figure 4). The temperature peak of the exothermal effect of decomposition is also shifted from 250 °C (p-(GAT-*co*-GA)-75) to 266°C (p-(GAT-*co*-GA)-14). Compared to azide homopolymer GAP, the p-(GAT-*co*-GA) copolymers exhibit a higher thermal stability (Table 2).

The density of all the powder-like polymers was measured by a helium pycnometer at 28.8 ± 0.8 °C. The average density and standard deviation were estimated from the results of 10 experiments (Table 2). The experimental density of N-glycidyl-5-aminotetrazole polymer (p-GAT) is 1.436 g/cm^3^. The transformation of the p-GAT homopolymer by nitration ensures a considerable density increase to 1.540 g/cm^3^. For the p-(GAT-*co*-GA) azide copolymers, the density value varies from 1.427 g/cm^3^ (p-(GAT-*co*-GA)-14) to 1.413 g/cm^3^ (p-(GAT-*co*-GA)-22) according to the ratio of the tetrazole and azide moieties. The polymers functionalized by 5-AT, completely (p-GAT) or partially (p-(GAT-*co*-GA)), demonstrate an enhanced density compared to the structural azide analog GAP whose density is 1.284 g/cm^3^ [5]. The standard deviation in estimating the density of all the polymers under study was not above 0.0008 g/cm^3^.

The nitrogen content of the resultant compounds varied from 42.26 to 47.95% and depended chiefly on the content of tetrazole heterocycles in the polymeric chain structure (Table 2).

The analysis of the findings demonstrates that the aminotetrazole heterocycles incorporated into the polyglycidyl chain provide high energetic (density) and thermal characteristics of the polymeric material. The level of the said properties can be tailored and controlled by varying the content of tetrazole moieties in the macromolecule. The nitrated p-GAT-N sample, despite having a satisfactory level of thermal stability, exhibits quite a high nitrogen content and an enhanced density compared to all the substrates studied herein.

### 3.3. Viscosity Characteristics and Curing Rheokinetics

The viscosity characteristics and rheokinetic parameters of curing were examined on samples of the p-(GAT-*co*-GA) azide copolymers and the p-GAT homopolymer as solutions in a composite tetrazole plasticizer, N-(2-hydroxyethyl)tetrazole (HET), consisting of N-1 and N-2 substituted isomers in a ratio of 30.3/69.7% (as per gas chromatographic data). For this, the corresponding polymer was mixed with the HET plasticizer in a ratio of 50:50 wt.%, and the resultant mixture was held at 50 °C for 15 h, then the solution was cooled to 25 °C and analyzed. Upon a prolonged storage of the prepared binary systems, no phase separation or any other signs of inhomogeneity were observed.

**Figure 5:**
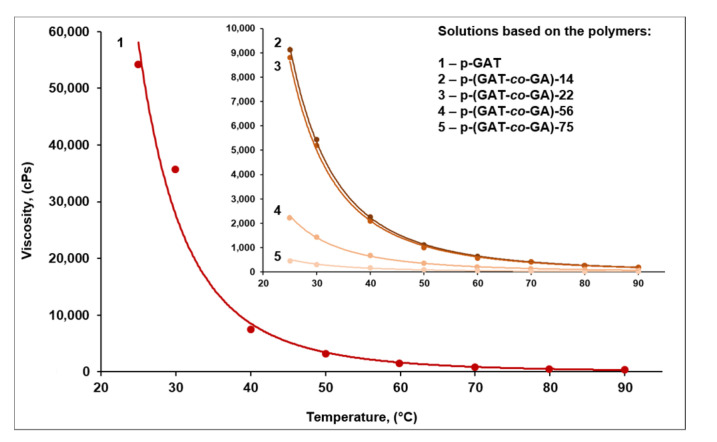
The viscosity of the p-GAT and p-(GAT-*co*-GA) solutions in the HET plasticizer plotted against temperature.

The temperature-dependent viscosity was evaluated in a range of 25–90 °C. For all the samples examined, the dependence was the same kind, that is, the expected decline in viscosity as the temperature was raised (Table 3, Figure 5). The greatest change in viscosity of the samples occurred between 25 °C and 50 °C, and the viscosity level diminished to a lesser extent above 60 °C.

The solution viscosity heavily depended on the ratio of the aminotetrazole and azide moieties contained in the polymeric matrix structure. At an equal temperature value, the binary system based on the p-GAT aminotetrazole homopolymer had the highest viscosity (Table 3). The highly polar bulk substituents (5-AT heterocycles) found in the side chain of the homopolymer macromolecule promoted a considerable increase in viscosity of the system based on this polymer. An increase in the content of N_3_ groups had an inverse effect and led to a decline in the dynamic viscosity of the solution because the azide moieties of the polymer acted as an intramolecular plasticizer and significantly increased the overall flexibility and mobility of the macromolecule.

**Figure 6:**
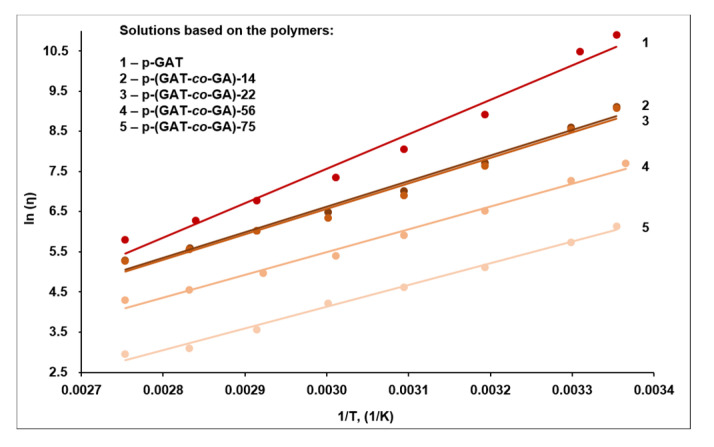
A plot of *ln* (η) versus inverse temperature (1/T) for the p-GAT and p-(GAT-*co*-GA) solutions in the HET plasticizer.

Using the data obtained for the temperature-dependent viscosity (Figure 5), we built a graphical plot of *ln* (η) versus temperature (1/T) (Figure 6), and the activation energy of the viscous flow (E_a_) for the respective sample was determined from the slope of line, with the Arrhenius log Equation [77] factored in. An augmentation of the azide segments in the polymer structure promoted a decline in the activation energy required to overcome the flow resistance arising from intermolecular forces (Table 3).

**Scheme 2:**
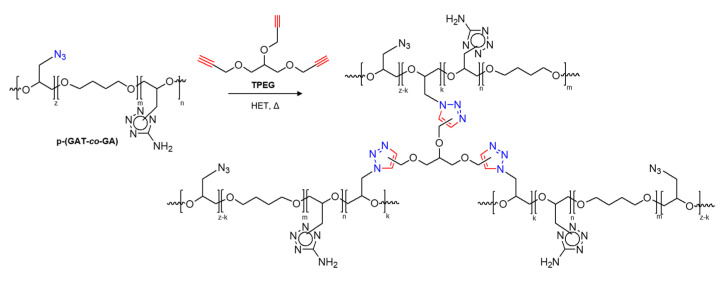
A scheme of reaction between the p-(GAT-*co*-GA) copolymers and TPEG.

The reaction of 1,3-dipolar cycloaddition between the N_3_ groups of the copolymers and the propargyl groups of the trifunctional acetylene, 1,2,3-tris(propyl-2-ynyloxy)propane (TPEG) was used to assess the rheokinetic parameters of the initial stage of the curing process of p-(GAT-*co*-GA)-based binary systems. This reaction (the so-called click-chemistry reaction) is offered as an alternative to the isocyanate curing method of GAP, widely employed to generate chemically and thermally stable 1,2,3-triazole bridges and obtain cross-linked polymeric chains [78,79]. The formation of spatially branched energy-rich polymeric binders comprising both 5-aminotetrazole and 1,2,3-triazole heterocycles is illustrated in Scheme 2.

The curing process was run at 80 and 60 °C, with varied ratios of –C≡CH/N_3_ groups and without catalysts.

As a result of thermal cyclization, the functional groups of polarophilic TPEG coordinate with the azido groups of the copolymer to furnish 1,4- and 1,5-substituted triazole heterocycles (Scheme 2).

**Figure 7:**
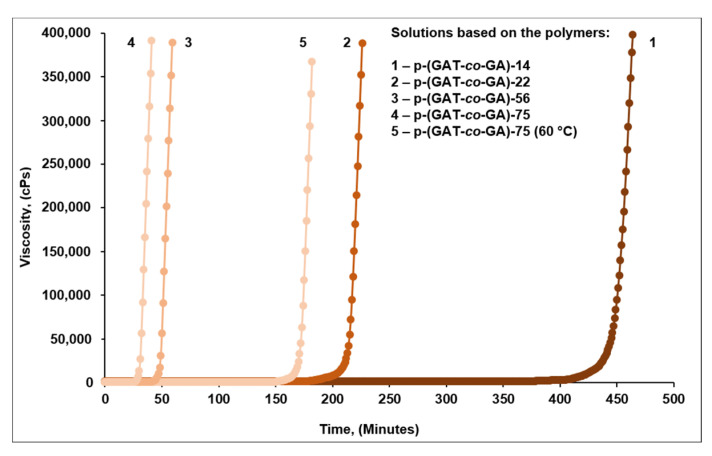
A time course of the viscosity of solutions based on p-(GAT-*co*-GA) copolymers when cured at 80 °C (60 °C) (a constant shear rate of 0.05 s^−1^).

The rheokinetic parameters of the initial reaction stage of 1,3-dipolar cycloaddition (Table 3) were measured from the change in viscosity over time (rheokinetic curves, Figure 7) and from the change in the absorption band intensity of the N_3_ groups (near 2100 cm^−1^) or its complete disappearance in the IR spectrum.

**Figure 8:**
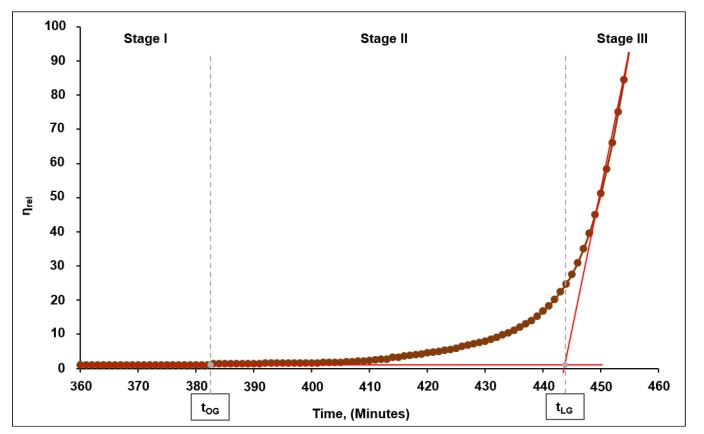
A typical rheokinetic curve of curing of the solution based on the azide copolymer, as exemplified by p-(GAT-*co*-GA)-14.

It is seen from the typical rheokinetic curve of curing (of the p-(GAT-*co*-GA)-14-based binary system) depicted in Figure 8 (where the relative viscosity (η_rel_) represents a relation between the viscosity of the system at the *i*-th moment of time and the original viscosity) that the reaction between the azide groups located in the polymeric side chain and the propargyl groups of the TPEG curing agent occurred successively in three stages.

Stage I was the gradual formation of branched macromolecules, with the formed structures retaining the abilities to transit into the viscous-flow state or be solubilized. A slight increase in the viscosity of the system over time at this stage was due to a linear rise in the molecular weight of the polymeric binder because of a partial addition of the TPEG molecules.

The aforesaid stage continued until the onset of gelation (point t_OG_ in Figure 8) and is illustrative of the viability time or so-called survivability of the binary system.

Stage II is a transition stage from the onset of gelation to the so-called transition point of gelation (point t_LG_ in Figure 8), at which a more intense increment in viscosity over time was observed when compared to the first stage.

**Figure 9:**
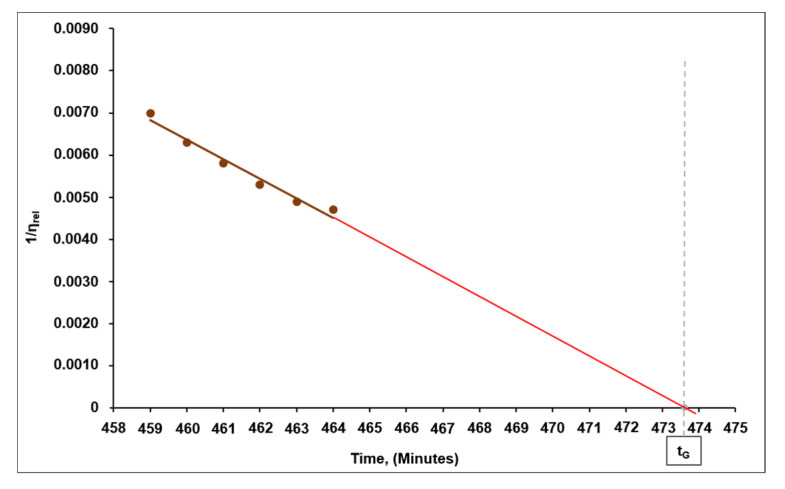
Gelation point (t_G_) measurement, as exemplified by the solution of the p-(GAT-*co*-GA)-14 copolymer.

Stage III involved a further conjugation between the branched macromolecules and a final formation of spatially branched (chemically cross-linked) 3D structures. That said, a sharp increment in viscosity of the system was observed over time from point t_LG_ to the point of gelation t_G_ (pour point), while the polymeric binder lost its capabilities of solubilization and transition back into the viscous-flow state. The characteristic transition point of gelation (t_LG_) was determined by intersection of the extrapolating lines built for the initial, almost horizontal region of stage I and for the final region of stage II, wherein an appreciable increase in viscosity is observed (Figure 8). The time of gelation (t_G_) was determined by extrapolating the relationship between the inverse value of the relative viscosity (1/η_rel_) and curing time towards zero, that is, the infinitely great value of relative viscosity at which the system reaches the pour point (Figure 9).

**Figure 10:**
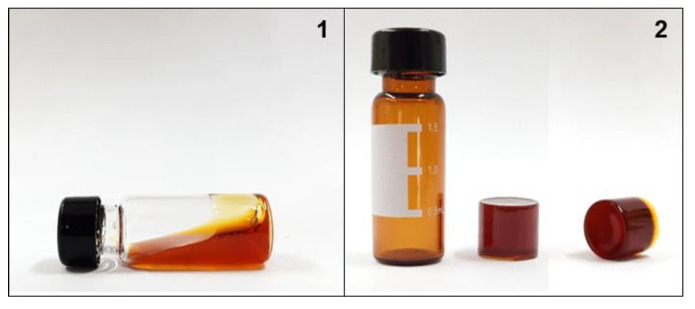
A sample based on the p-(GAT-*co*-GA)-14 copolymer before (**1**) and post-curing (**2**).

Finally, the system gradually transits from the original viscous-flow liquid (Figure 10, 1) to the cured vulcanizate during the process (Figure 10, 2).

Depending on the content of azide moieties in the copolymer macromolecule and the curing temperature, the onset of gelation (“survivability”) can be controlled over a wide time interval (Table 3): from 26 min (for p-(GAT-*co*-GA)-75) to 383 min (for p-(GAT-*co*-GA)-14) and from 26 min (at 80 °C) to 151 min (at 60 °C).

## 4. Conclusions

Here, we reported the results for the potential synthetic feasibility of the idea towards novel, promising, energetic polymers based on 5-aminotetrazole and investigated a range of basic characteristics thereof. The findings demonstrate that the p-GAT homopolymer and p-(GAT-*co*-GA) azide copolymers exhibit a high density and a high nitrogen content and are highly thermostable compounds. The nitrated p-GAT-N polymer comprises additional energy-rich elements, has a satisfactory thermal stability and quite a high nitrogen content and possesses an enhanced density among the polymers developed herein. For the p-(GAT-*co*-GA) copolymers, their potential application as a scaffold of energetic binders was additionally evaluated.

Overall, the findings from this study demonstrate that the modification of GAP through complete or partial hypothetical replacement of azide groups by 5-aminotetrazole heterocycles and their nitrated derivatives holds promise. Such a high-density nitrogen-rich thermostable material can serve as a promising energy-rich polymeric binder to make high-performance energetic materials.

## Figures and Tables

**Table 1:** Synthesis data and structural characteristics of the p-(GAT), p-(GAT-*co*-GA) and GAP polymers.

Polymer	Reactant Ratio, ^a^ mmol		Element Ratio, ^b^ % mol		Ratio of Isomeric Moieties, ^c^ % mol.		M_w_^d^	M_w_/M_n_^d^	Yield, %
	Na5-AT	NaN_3_	GAT	GA	N-1	N-2			
p-GAT (homopolymer)	150	0	100	0	42.9	57.1	5569	1.45	52
p-(GAT-*co*-GA)-14	135	15	86.1	13.9	43.8	56.2	4041	1.33	57
p-(GAT-*co*-GA)-22	125	25	77.7	22.3	45.7	54.3	3329	1.31	62
p-(GAT-*co*-GA)-56	90	60	44.1	55.9	48.9	51.1	2642	1.28	71
p-(GAT-*co*-GA)-75	75	75	25.4	74.6	50.8	49.2	1643	1.26	61
GAP (homopolymer) ^e^	0	150	0	100	–	–	1303	1.19	74

^a^ The ratio of the reactants in the synthesis of polymers. ^b^ Calculated by ^13^C NMR spectroscopy. ^c^ Calculated by ^1^H NMR spectroscopy.^d^ Determined by GPC (DMF, PEG standards). ^e^ GAP was prepared by the procedure reported in [23].

**Table 2:** Measurement results of thermal stability and density of polymers.

Polymer	T_dec_ (onset), °C	T_dec_ (max.), °C	Density, g/cm^3^	N Content, %
p-GAT (homopolymer)	243	272	1.436 ± 0.0002	47.95
p-GAT-N	122 241	145 278	1.540 ± 0.0008	40.10
p-(GAT-*co*-GA)-14	233	266	1.427 ± 0.0002	46.98
p-(GAT-*co*-GA)-22	229	263	1.413 ± 0.0002	46.23
p-(GAT-*co*-GA)-56	225	254	viscous-flow	43.81
p-(GAT-*co*-GA)-75	220	250	viscous-flow	42.26
GAP (homopolymer) ^a^	200	246	1.284	40.95

^a^ From Ref. [3].

**Table 3:** Viscosity characteristics and rheokinetic parameters of curing (at 80 °C) of the polymers plasticized with HET (in the form of 50% solutions).

Polymer	η, ^a^ cPs at 25 °C	η, ^a^ cPs at 60 °C	η, ^a^ cPs at 90 °C	E_a_,^b^ kJ/mol	t_OG_, ^c^ min	t_LG_, ^d^ min	t_G_, ^e^ min
p-GAT (homopolymer)	54249	1551	333	71.2	–	–	–
p-(GAT-*co*-GA)-14	9126	653	200	52.9	383	444	474
p-(GAT-*co*-GA)-22	8816	568	194	52.6	183	214	231
p-(GAT-*co*-GA)-56	2220	221	73	47.2	41	48	65
p-(GAT-*co*-GA)-75	460	68	19	45.1	26 151 ^f^	30 172 ^f^	46 188 ^f^

^a^ Dynamic viscosity. ^b^ Activation energy of viscous flow. ^c^ Onset time of gelation. ^d^ Threshold time of gelation. ^e^ Gelation time. ^f^ Value at 60 °C.

## Data Availability

Not applicable.
